# Discourse on African American/Black Identity: engaging the expanded nigrescence theory with a diasporic consciousness

**DOI:** 10.1186/2193-1801-2-233

**Published:** 2013-05-24

**Authors:** Patrick S De Walt

**Affiliations:** Psychological and Social Foundations, College of Education, University of South Florida, 4202 East Fowler Avenue –EDU105, Tampa, FL 33620-5650 USA

## Abstract

This manuscript theoretically explores the application of a stretched expanded nigrescence theory (NT-E) by making notions of consciousness explicit, thereby building on the research involving first generation U.S.-born Africans (FGAs). In taking this approach, a theoretical framework of a diasporic consciousness emerged an alternative for exploring the identity development of Africana people. To facilitate this process, a shift from conceiving identity along the lines of race and ethnicity was begun towards a conception that is solely culturally based on eight identified factors.

The process Africana people have used to self-identify individually and collectively has gone through much iteration. Cross ( 1971,1991,1995 ; Vandiver et al. 2002 ) is, perhaps, one of the most widely known and researched of these iterations. Cross’s ( 1971 ) original formulation (NT-O), revised version (NT-R) ( Cross 1991 ) and the subsequently expanded nigrescence theory (NT-E) ( Cross and Vandiver 2001 ) suggest that Black identity development results from a progression through levels of racial consciousness and identification demonstrated in response to political awareness and/or forms of oppression. Cross, Grant, and Ventuneac ( 2012 ) suggest that “Nigrescence Theory presupposes there is not a single form or type of black identity and that a large sample of black adults reveals a broad range of identity orientations, resulting in a classification challenge” (127).

Furthermore, Cross and Vandiver ( 2001 ) identify several premises regarding Nigrescence theory, within this discussion, the focus is on two particular areas in which the authors address:1) “that there is more than one type of Black identity, which results in the delineation of a range of identity exemplars… [and 2)] that great variability exists in the way Black people make meaning of and interpret their social sense of self” (380–181). With this “classification challenge” and the emphasis on these premises in mind, De Walt ( 2009 ), proposes that the NT-E as currently formulated does not account for the identity development of several groups of Africana people such as first generation U.S. born Africans (FGAs). This position regarding FGAs’ self-perceptions of their identities relates to notions of Blackness that are fundamental to the theory itself.

It should be argued that what “Blackness” traditionally means, within the conception of NT-E, is situated in the discourse around black identity as experienced and enacted in the U.S. context which translates from the Black Power period’s clear delineation from prior impositions that promoted senses of inferiority, instead for purposes of empowerment Ogbar ( 2004 ). In taking this fluid conceptualization of “Blackness” into consideration, from a U.S. perspective, the cultural representation of “Blackness” as considered within the discourse still has foundational tenets within it that are situated in the “Black struggle” as experienced within the U.S. first and foremost as opposed to something that is truly Pan-African. This historically significant factor can be seen in the many ways that the Africana globalized struggle has made use of the struggle as it has evolved within the United States. This implicitly creates a cultural disconnection between members of the diaspora when applying U.S. centered frameworks that are based on U.S. Black Nationalistic norms as opposed to those that offer a more globalized perspective. For this reason, as it relates to those who are culturally connected yet septed as what Du Bois ( 2003 ) references in double consciousness as a “two-ness” that manifests itself culturally. With this in mind, Blackness can be describes in terms of “Black identities” as Rahier ( 1999 ) states, “are defined and redefined, imagined and re-imagined, performed and performed again within the flux of history and within specific, changing, spatially determined societal structures” (xxiv). The intent of this paper is to explore how NT-E could be further expanded by illuminating NT-E’s implicit connections with early and more recent conceptions of Africana consciousness, which includes a more inclusive depiction of Blackness or Africanity, as a means of ultimately increasing the inclusiveness of NT-E to all Africana peoples.

This article extends the discussion regarding the augmentation of the NT-E, which began in De Walt ( 2009 ) as a result of the application of NT-E, to a population of FGAs as opposed to Generational African Americans (GAA), children of African Americans born in the U.S., attending a predominantly white institution (PWI) of higher education. This paper continues to investigate the features that contribute to the racial identities and attitudes of Africana people particularly as related to the concept of Blackness as applied within the theory as enacted and perceived in a U.S. context. The discussion will then turn to Diasporic Consciousness, a theoretical framework that works with NT-E, and the concepts that underpin this conception with a focus on culture.

## Racial identity development and consciousness

### Racial identity development

Racial identity development of Africana people has been discussed by a number of researchers ( Akbar 1989 ; Cokley 2002 ; Cross 1971,1991,2012 ; Cross 1978,2001 ; Cross and Vandiver 2001 ; Helms 1990 ; Nobles 1989 ; Sutherland 2011 ; Vandiver et al. 2002 ; Worrell, Cross and Vandiver Cross and Vandiver 2001 ). Worrell, Vandiver, Schaefer, Cross and Fhagen-Smith ( 2006 ) examined how well the nigrescence profiles generated by using the Cross Racial Identity Scale (CRIS) would match other identity characteristics of an Africana population at a PWI and at a historically Black college (HBCU).

Although Worrell et al. ( 2006 ) support the premise that the CRIS takes when engaging NT-E in regard to the referent group orientations (RGOs) of some Africana students, this recognition of Blackness is not inclusive of all forms of Africanity within the U.S., particularly those who are categorized as African American or Black by the U.S. Census and higher education. While Blackness, in the U.S. context, historically is both a political and cultural identity marker, within its use within nigrescence, it does not appear to be inclusive enough in its efforts to capture alternative expressions of “Black” identity. This reality is evidenced through the narratives of FGAs ( see De Walt 2009 ) indicating that this framework seems primarily based on fundamental facts and periods of U.S. history. Findings from Worrell et al. ( 2006 ) support the position taken within this paper that the NT-E is not yet fully inclusive.

There is little research exploring identity development of Africana people who are descendants of those enslaved beyond the current constructs of race and ethnicity within the U.S. The following studies position themselves in ways that are linked to race and ethnicity but align more so with a cultural identity framing. Bailey ( 2001 ) and Saucedo ( 2002 ) sought to reject outright that notion that a Black identity existed universally. Bethea ( 2006 ) and Hall and Carter ( 2006 ) also found little support for U.S. conceptions of being “Black” among non-U.S. born African populations. Bailey ( 2001 ) identifies the challenges associated with Black racial identification as well as the rejection of notions of being “Black” by over 30 Dominican-American high school students between the ages of 16–18. Bailey ( 2001 ) found that Dominican-Americans strategically embraced the identity labels “Spanish/Hispanic/Latino” and rejected “Black” identity as well as absorption into a U.S. racial hierarchy where a large number of Dominicans were excluded from the category of White. In terms of their identities, this population chose to self-identify in a manner that does not fit into the racial dichotomy of Black and White historically and legally constructed in the U.S. regarding race as visible phenotypes ( Davis 1993 ; Harris 1995 ; Gordon 2000 ; Mills 1998 ). This is important to note as they personify what many other Africana people within the U.S. contend ( De Walt 2009,2011 ; Romo 2011 ) based on an array of cultural enactments (e.g., language).

Hocoy ( 1999 ), who studied racial identity development in South Africa, offers three important features when discussing racial identity outside of the U.S. These are: the unique importance and salience of race in the country, the greater degree of societal discrimination based on race, and South Africa’s indigenous African context and the omnipresent influences of African heritage” (131). These three areas are important to acknowledge when addressing the continental African elements that are a part of FGA identity.

Discourses on immigrant populations underscore significant points that coincide with those of FGAs who are themselves children of immigrants. The works of Waters ( 1994,1999 ), Ogbu ( 1991,1992 ), Rumbaut ( 2002 ) and others highlight the experiences of Black immigrant populations who migrate to the U.S. and are subjected to the experiences associated with Black identity. The significance of encounters with the stereotypes of GAAs and Black Americans were also identified via the experiences of West Indian populations ( Waters 1999 ).

### Stretching the nigrescence theory (NT-E)

**Figure 1:**
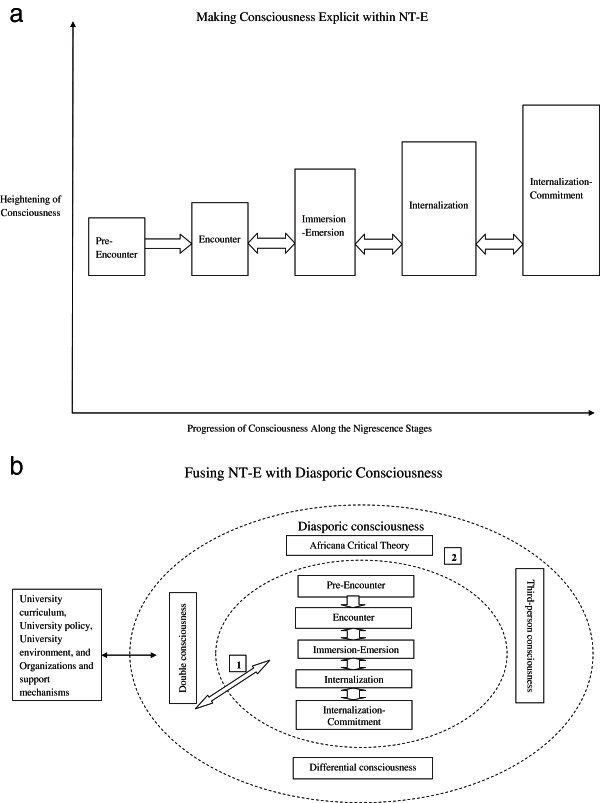
**Engaging NT-E with Notions of Consciousness.a**. Making consciousness explicit; **b**. Fusing NT-E with Diasporic Consciousness.

Cross ( 1991 ) states that everyone who has a Black identity may not be Afrocentric,…and Afrocentricity does not incorporate all legitimate interpretations of Blackness (222).” Cross’s observation was one of the factors that motivated De Walt’s ( 2009 ) study of FGAs. In making this assertion, Cross’s discussion of Blackness within all of his iterations of Nigrescence (NT-O, NT-R, NT-E) (1971, 1978, 1991, 1995, 2013) was not only focusing on Black Nationalism but a particular variant of Black Nationalism, one fashioned by occurrences within the U.S. The results of this study confirmed Cross’s earlier assertion. Accordingly, there seems to be a need to expand NT-E so that it incorporates an African diasporan perspective while clearly identifying the theoretical elements that are embedded within its design (see Figure 1a).

Cross ( 1991 ) embodies elements of the theories of both Du Bois and Fanon within his conceptualization of nigrescence and was also significantly influenced by Fanon’s ( 1963,1967a 1967b ) “thinking about racial or cultural identity development and liberation struggles” ( Sneed et al. 2006 :70). Cross ( 2012 ) furthers this argument as he clarifies his intentions with nigrescence and the CRIS in regards to race, noting that his “work has always been about racial identity as oppression *as well as* racial identity as culture” (xvi). With this thinking in mind, he continues on by stating, “blackness is far more ethnic-cultural and existential than ‘racial,’ it may come as a surprise to the reader that I experience a certain degree of discomfort when one refers to my work as solely as ‘racial’” (Cross:xvii). These points require mentioning as I situate my argument for the facilitation of a theoretical shift that is not shared by the theorist whose work is critical to my theoretical exploration. Cross clearly argues that, if racial and cultural identity were totally distinct constructs (which, of course, they are not), adequate psychological functioning requires development of both domains and that in combination (connectivity), one is afforded the best defense against internalized oppression and good coping in the face of race and related stress (xix).

When engaging aspects of Africana people’s identities that have been racialized and ethnicized, there needs to be a distinction between how we see each other as opposed to the means in which those identities are both racially and ethnically altered in ways that perpetuate those oppressive structures that historically exist. This is the challenge that Cross argues in terms of his perspective of a “racial-cultural socialization”.

Worrell ( 2012 ) provides a review of nigrescence theory over a forty year period. Within this work, he states “a new direction for NT-E” (15). Within this discussion, he describes the development and implementation of the CRIS for this purpose. What is most important in Worrell’s discussion of nigrescence, as it relates to this current presentation, focuses on his acknowledgment of “a need in the literature for an instrument that allows us to conduct comptive and cross-cultural studies of cultural identity in a more nuanced fashion” (16). In thereby recognizing the nuances found within cultural identity, Worrell furthers the theoretical objectives of this article as it seeks to illuminate the multiple enactments of Africana people’s cultural identity.

#### Figure 1a making consciousness explicit

This diagram further represents the element of consciousness that is inherent within the NT-E as presented by Vandiver et al. ( 2001 ). Within this conception, consciousness is heightened as one moves forward in this process of traversing the nigrescence stages. The augmented NT-E, as described within this presentation, moves to the center of this discussion by taking key elements of what was developed over time by Cross ( 1971,1991 ) and then in collaboration with Vandiver ( Cross and Vandiver 2001 ) to create an identity model that would operate in a more robust manner in connection with the CRIS. This conceptual framing also aims to address areas of contention by others towards Cross and Vandiver’s framing of NT-E around issues and concerns of Westernization and Afrocentrism ( Akbar 1989 ; Kambon 1992 ; Nobles 1989 ). In response, Vandiver, Fhagen-Smith, Cokley, Cross and Worrell ( 2001 ), stated that “advancing…Black identity development is best served by a healthy interaction between theory and research” (199). This statement supports the presentation of an augmented theoretical framing of the NT-E that navigates the fairly new area of identity development for FGA students.

### Perspectives on consciousness

Perspectives on group consciousness that are maintained through a Pan-African lens and experience allow for the regionalized effects of culture and language to be acknowledged thus providing space for heterogeneity. The concern with NT-E is that the traditional markers used to attain reference group orientation (RGO) do not fully engage the cultural expressions of FGAs and other Africana identities ( Hocoy 1999 ). The proposed augmented framework is comprised of elements drawn from Africana critical theory ( ACT; Rabaka 2010 ), double consciousness ( Du Bois 1986 ), third-person consciousness ( Fanon 1967a ) and differential consciousness ( Sandoval 2000 ). Synthesis of these forms of consciousness intend for an application of Pan Africanism that is not relegated to the conception solely derived from elements framed within the U.S. context.

#### Africana critical theory

The work of Rabaka ( 2002,2006,2007,2009,2010 ) provides an important means for grouping aspects of Africana experiences while simultaneously acknowledging the various strands that comprise Africana people and culture. Africana critical theory is “*theory critical of domination and discrimination in classical and contemporary, continental and diasporan African life worlds and life struggle*” ( italics by Rabaka 2010 :14).

Within the context of this exploration and overall cultural identity of Africana people, inherently by its design, ACT engages all aspects of this theoretical effort in a global/transnational manner. By synthesizing aspects of ACT’s multidimensionality and embedding them within each of the components of not only diasporic consciousness but NT-E as well, aspects of its classical and contemporary mission can be achieved. The significance of ACT’s versatility becomes clear as seen in the circular nature of the theory and its theoretical extension beyond Cross’s ( 1991 ) use of Afrocentricity. Africana Critical Theory includes the notions of region and place that Asante ( 1988,1998 ), Cabral ( 1973 ), Kambon ( 2012 ), and Nobles ( 2006 ) articulate.

De Walt ( 2009 ) suggests notions of Afrocentricity identified by Cross contain a historical and U.S. Black national articulation. This articulation of Afrocentricity is incomplete, in part, due to the language, as exemplified in the CRIS ( Cross and Vandiver 2001 ; De Walt 2009 ). As a result of Afrocentricity as defined by Cross being born out of the Black Power Period, this ideology was centered in a U.S. context as opposed to a universalized Pan-African or Africana perspective. Accordingly, the identities represented by such groups as FGAs are not fully recognized in Cross ( 1991 ; Cross and Fhagen-Smith 2001 ) articulation of Afrocentricity.

#### Double consciousness

There is a need for a level of consciousness that captures the historical duality of FGAs and GAAs within U.S. society. This is, perhaps, best described by W. E. B Du Bois’s notion of double consciousness, "the sense of always looking at one’s self through the eyes of others, of measuring one’s soul by the tape of a world that looks on in amused contempt and pity. One ever feels… two-ness…two thoughts, two unreconciled strivings; two warring ideals in one dark body" (1986:364–5).

Double consciousness represents a dilemma that most GAAs face in today’s society where the manner in which they can represent their cultural identities may be placed at odds based on currently accepted norms of what constitutes Black identity within the U.S. Furthermore, all Africana people in the U.S. live with these false phenotypic and genotypic conceptions of Blackness that may not incorporate their traditional ways of being.

#### Third-person (triple) consciousness

Frantz Fanon ( 1967a ) suggests that consciousness of the body for people of African descent is “solely a negating activity [because] it is a third-person consciousness” (110–111). Fanon explains this by noting, “I was given not one but two, three places…I existed triply….I was responsible at the same time for my body, for my race, for my ancestors” (112).

Within Fanon’s own analysis, there are notions of nigrescence (the Pre-Encounter stage) potentially at work. Fanon’s conception provides a valuable insight that addresses not only the ideological sense of self that Africana people face within the U.S. and its universities, but also the physical natures by which their identities are fractured.

#### Differential consciousness

Sandoval ( 2000 ) provides a “four-phase hegemonic typology” of feminism as she extends both Du Bois ( 1986,2003 ) and Fanon’s ( 1967a ) notions of consciousness. She uses “differential consciousness” as a conceptual framework for understanding, and explaining varying modes of consciousness that seemingly appear to be antagonistic or in opposition to one another. Sandoval explains that differential consciousness is “linked to whatever is not expressible through word. It is accessed through poetic modes of expression: gestures, music, images, sounds, words that plummet or rise through signification to find some void—some no-place—to claim their due” (140). This “no-place” is the position that Africana people inhabit within White racist society and institutions in which their beings, fused or not, are in a state of flux or in a void as the other.

Differential consciousness allows for the consciousness of these perceived doubled and tripled identities of individuals and groups to engage various social situations differently and at multiple intervals. This perspective also requires the integration of ACT. This results from Sandoval’s ( 2000 ) use of Fanon’s ( 1967a ) work within the development and use of her theory. This application is not meant to diminish or deny Sandoval’s position and importance within the tradition of Chicana feminism and intellectual thought*.* Embracing this approach can offer agency as well as a strong sense of social position within racist environments. Each aspect of a FGA’s identity has the opportunity and the power to create psychological spaces in which she or he can recharge or re-strategize their self-concept in order to manage hostile situations.

Another form of empowerment is hereditary history. Double consciousness makes use of this *collection of denied historical and ancestral memories resulting from the lived experiences that are transmitted generationally among oppressed groups in efforts to reclaim a wholeness of self through resistance to colonialism.* In this way, the use of hereditary history is a prime example of the empowerment that ancestors provide through memories, hymns and historical images that take one into a different social and psychological space ( De Walt 2009 ; also see Du Bois 1986 ; Kombon 2012 ; Nobles 2006 ).

As a conceptual bridge between both double consciousness and triple consciousnesses, it serves a similar purpose as bridging does for Black identity when engaging other cultural identities, in particular White identity. This significance is stated by Cross ( 1991 ), “Keep in mind that because ‘Black-[W]hite’ conflict is at the core of nigrescence experience, the initial focus of bridging may be [W]hite society, [W]hite organizations, and the reestablishment of [W]hite friendships” (218).

#### Diasporic consciousness

Diasporic consciousness inherently seeks to include all of those who are members of the diaspora and to accept their identities as they exist. Although Afrocentricity begins this process, it also excludes some identities whereas ACT is more open to these possibilities. Diasporic consciousness is *a heightened awareness drawn from synthesizing elements of Africana critical theory (Rabaka*^2010^*), double consciousness (Du Bois*^1986^,^2003^*), third-person (or triple) consciousness (Fanon*^1967^a *) and differential consciousness (Sandoval*^2000^*) within varying colonial contexts that is sociological, culturally/linguistically, politically, historically, and spiritually/religiously grounded for the purposes of the psychological, political, sociological, and economic liberation and agency of diasporan communities through reclamatory humanism* (De Walt 2009 ). Reclamatory humanism is a systematic process by which marginalized or oppressed groups recapture and reclaim their humanity through liberating acts that are economically, educationally, politically, religiously/spiritually, and/or socially/communally-based ( De Walt 2009,2011 ). Cabral ( 1973 ) articulates this sentiment when stating, “look up these [African cultural] values as a conquest of a small piece of humanity for the common heritage of humanity, achieved in one or several phases of its evolution” (51). Elements of each of these four concepts (Africana critical theory, double consciousness, third-person consciousness and differential consciousness) that constitute a diasporic consciousness are applied to this particular group and context, FGAs attending PWIs ( De Walt 2009 ).

#### Figure 1b fusing nt-e with diasporic consciousness

The identification and application of a diasporic consciousness is highlighted through the illumination of a heightening of consciousness within the NT-E (see Figure 1a). This articulation was used successfully to make diasporic consciousness explicit while engaging nigrescence and identity development at a PWI. This cellular-like illustration in Figure 1b provided ways of grasping the complexity found within the Africana identity development of those who, in particular, are FGA and attend a PWI. It illustrates how various stimuli impact FGAs at various levels of their consciousness.

A conceptual framing of the stretching of the NT-E involves identifying the elements of a diasporic consciousness that simultaneously work with the traversing of four of the five identity stages as well as recognizing the existence of a different type of Black identity, the FGA identity. Cross and Fhagen-Smith ( 2001 ) discusses the idea of “stretching” NT-E theory to include children such as those described by Tatum ( 1997 ). This attempt of stretching his theory is meant to be independent of Tatum’s use of Cross’s theory. In other words, diasporic consciousness plays a vital role in the identity shifts that take place within the revised identity model presented by Cross. This is meant to further highlight the potential ways in which the socializing elements impact these identity shifts by FGA students. This approach also has the potential of operating within the notions of “recycling” as identified by Parham ( 1989a 1989b ) and Cross ( 1991 ). De Walt ( 2009 ) elucidates the ways in which this theoretical exploration (see Figure 1a-b) engages the concept of recycling in which not only Parham ( 1989a 1989b ) but also Cross and Fhagen-Smith ( 2001 ) engage in terms of nigrescence.

This diagram also introduces four particular types of stimuli that can impact the identity layer (1). These include: (a) university curriculum, (b) university environment, (c) university policy and organizations, and (d) support mechanisms that act to highlight the various socializing elements of the institution. These are activated when a student’s consciousness is raised through experiences that are perceived to be racist, discriminatory or prejudicial. There is an extensive body of literature ( Brown et al. 2007 ; Davis 2004 ; Feagin et al. 1996 ) not limited to Africana people, that addresses how these items impact the learning and social development of students at the university level.

The black arrow represents how these four particular items enter the perforated layers to sometimes reach the identity represented by layer I. The white arrow represents how the identity shifts (layer 1) are potentially impacted by layer 2 (diasporic consciousness) and vice-versa. Diasporic consciousness occupies Level 2 and operates as a cellular wall/membrane serving as a protective barrier for the core identity or nucleus (layer 1). This manifestation acts along the lines of a cellular-like structure providing permeability, negotiating the interactions with external forces or stimuli as represented by the black arrow. When looking at diasporic consciousness (layer 2), it is important to notice the perforations are meant to show that it is not impenetrable and that impacts are bi-directional. This also opens the possibility to how (diasporic) consciousness works, depending on the level of heightening/awareness, to impede or allow (i.e., “selective permeability”) these various stimuli to reach the core identity or nucleus ( Campbell and Reece 2005 ).

The black arrow is also meant to show a varied ability of these items to influence a person throughout the stages of the core of this identity structure. This also shows the ability of the various stages of the core identity to influence these stimuli as well. This understanding also creates opportunities for the actualization of individual and/or communal agency among, in this discussion, FGA and GAA communities/individual members. One example of this type of agency often discussed regarding nigrescence, in the 1970s, was the development of black/(Generational) African American Studies programs and departments across the U.S.

### Implications for policy and practice

#### The university climate

The theme of diversity remains a core component of what I offer as implications for research and practice. Without this notion of diversity, a heterogeneous perspective on Africana people cannot occur. In saying this, much of the experiences of FGA students (De Walt 2009, 2011) centered on the university climate as it is promoted or at least not challenged. The ideas of having students who are nothing more than window dressing for the benefit of others defies any humanistic logic. The University issues much in its rhetoric but little in its concrete day-to-day actions that students can grasp and incorporate from their frames of reference. This frame of reference for FGAs is situated within identities of “Black” and “African American” and the perspectives held by others.

Recognizing the varied ways in which African American and/or Black identities are represented creates much needed discussions on the ways in which universities should include, teach and provide viable opportunities for the acknowledgement of difference among Africana people. These steps are not meant to be superficial, but authentic in design and delivery. Students recognized detached supportive means that were seen more for “cosmetic effect” than as effective, with an example being that of the University’s efforts regarding diversity. As a result, PWIs are not fully equipped to engage diverse populations in manners that do not appear to repeat some of the limits of Multicultural education practices that simply tokenize those of whom are different. Or instances where the *gentrification of ethnicity* prevails in which white students embrace their ethnic heritages thereby avoiding notions of whiteness while simultaneously maintaining the resulting privilege.

In saying this, the aims of the PWI should be to actively see diversity and culture as “embedded in context” as Sonia Nieto ( 1999 ) discussed in *The Light in Their Eyes: Creating Multicultural Learning Communities*. PWIs by their current structure have Eurocentric cultural components embedded within as a result of the make-up of the student population and much of the staff. This allows the norms of the majority to be the consensus regarding what occurs within the campus both as policy and at the community level. To offset this will require more than a cultural holiday or event sprinkled in to remind diverse students who are of those communities to feel a part of and appreciated by the institution. These thoughts have been promoted by many multiculturalists as they pursue an educational experience that is more communal and from which all cultures benefit.

The PWI must simultaneously rethink add-on programs that are detached from the core mission of the University because they maintain the “detached status” that many student populations may experience, such as FGAs, GAAs, Native Americans, Asian Americans, Mexican-Americans, Chicana/os, GLBTQ and many other cultural groups. The idea of having safe spaces for them and others to occupy is important, but it simultaneously isolates them in the process because most of the “safe places” if/when provided by the University and its support mechanisms are isolated themselves. Policy should require that support mechanisms be more in touch with the student populations through student liaisons, faculty, staff and the administration working together while collectively representing the populations they serve. Implementation of such frameworks as an expanded NT-E with a diasporic consciousness can help university staff better program for a diverse Africana community that does not solely prepare for students to fit within traditional U.S. centric forms of Blackness while not denying those who do fit within those frameworks access to valuable university programming (i.e., Multicultural Affairs/Programs). Sheer acknowledgment of their complex identities is also of need when it comes to serving diverse student bodies.

A shift is needed towards collectivism beyond the embracing of knowledge construction that is less culturally exclusive. It needs to include representation that is not seen as only a “cosmetic effect”, but that actively addresses the concerns of the study body in more meaningful ways. Is it an educational experience that is solely based on curriculum within the classroom? Or is it one that is meant to be all encompassing of the total human experience of each student? If it is the latter, then that mission of collectivism requires a monetary and moral investment that is tangible and concrete both in rhetoric and action. This occurs through forms of accountability that seek results but that also understand the reality of such contexts where the majority of students have relatively little interaction with those who are not like them prior to attending the University. This applies to both “students of color” and “White” university populations.

Also, a continued but honest effort to recruit faculty and students who are of diverse backgrounds needs to occur in a manner beyond just their phenotypes and genotypes that it often portrays. The University has situated itself as a staple of knowledge and social interaction, but it isolates many members of its community based not on how they look but also how they think. In a consumptive manner the University appreciates aspects that it deems worthy for the mission that it has designed. Culture is one such commodity that must not be used for the superficial purposes that are currently operating within the University.

Students such as FGAs recognize the emptiness of the policy and rhetoric that are often espoused by the University. Such cases highlight the lack of connection between the University and its policies and the students themselves. This remains a red flag that no amount of lip service can correct; the University must relieve itself of its false pretenses of accommodating the learner and promoting an inclusive, diverse^a^ community. For FGAs and students like them, it often reinforces the stereotypes and historical injustices that society still struggles to confront. Until then the University will remain a hollow entity for the purging of possibilities of students who are culturally, religiously, and/or linguistically marginalized^b^. Although the focus of this paper centers on Africana identity: FGAs and GAAs, all of these components of identity need to be incorporated within a university’s mission.

#### Identity labels

As the role of the University and/or its effectiveness has been questioned, there needs to be continued explorations of the ways in which the University and federal agencies label diverse populations. For many Africana students, they do not identify with some of the currently used identity labels, whether when having to apply for school or other social responsibilities where race is evaluated. This idea of the “Other” identity label that some universities use as a quick way of catching the loose identity strands is out of touch and sends a different message. “If you were written in, then were you ever truly considered?” This question is one that has historically been seen to manifest itself within every social movement within the U.S. that includes issues of suffrage and citizenship. From a perspective of consciousness, this question also shows that fundamental social differences remain as they are profoundly the foundation of U.S. society.

## Conclusion

This paper was undertaken as a result of the findings of De Walt ( 2009,2011 ), which indicated the need for an augmented NT-E. The concept of Blackness was one that many diasporan Africans in the U.S. see as a problematic term. For many of them, there is a negative stereotype or connotation to the term based on their interactions with others such as GAAs. In some of these cases, “Black” had an Anti-African component to it and consequently, it did not fully represent who they were. While there may be African Americans who have an anti-African perspective similar to what has been described above, for the sake of this study the views of some of the FGAs within this study viewed African Americans as those persons who embraced and sought to learn more about their African heritage. Resulting from these tensions, FGAs conception of Blackness often contradicted a foundational tenet of nigrescence. The process of self-definition for FGAs borrows from the GAA historical narrative while simultaneously challenging it on fundamental levels as notions of Blackness linked it with the continent and the Africana struggle for humanity. In acknowledging this occurrence within participants, their perspectives run counter to Vandiver et al.’s ( 2002 ) claim that RGO is different from personal identity in that RGO is based on their social affiliations preference, whereas personal identity refers to an individual’s sense of personal uniqueness (72). For some of the FGAs, their RGO and PI were similar in terms of the affiliations with their parents’ African heritages ( De Walt 2009,2011 ). This assumption that Black and/or African American identity is one that they willingly align within is also a point of contention as federal identity categories provide very few alternatives that fully affirm their identities.

Although this act on the part of “Blacks” may serve as an indication of the Pre-Encounter stage of nigrescence, these interactions over the course of the lived experiences of FGAs create an aversion to “Black” identity for them. Through this alternative understanding of these identities, FGA and GAA identities continued to exist in polarizing spaces. Also, this acceptance of being labeled by others creates an additional barrier between the process of self-determination and self-definition that is pmount to the social and political formulations of both the African American and Black identities within the U.S.

Nigrescence was complicated by the fact that much of what is its core was challenged by the existence of FGA identity. The idea of FGAs being poorly educated about the contributions of Africans to the world ideally should be viewed somewhat differently taking into account their African cultural backgrounds. For both Woodson ( 2006 ) and Cross ( 1991 ), what would be viewed as Black identity was positive but that was not what many of the participating FGAs have come to believe. This was also complicated because the participants who have this perspective were also the participants who have encountered Africana people who self-identified as Black, but also rejected any affiliation with the continent ( De Walt 2011 ). Therefore, these individuals took on an Anti-African positioning that impacted FGAs in a way that made them grow to think of being Black as being someone who does not want to associate with Africa.

This reasoning is also recognized by noting the FGAs’ continental African self, the African American self, which is the result of being born in the U.S. plus an historical view of that concept, and the American self or way of being that does not explicitly encompass African or African culture. This depiction of a triple-self appears to be an embedded Tri-Nationalism or Tri-Nationalistic identity ( De Walt 2009 ). As an extension of Fanonian thought, an embedded tri-nationalism or tri-nationalistic identity attempts to grapple with at least three forms of nationalism that possibly comprise FGA identity. A continental African self, an African American self and the American self or concept of being may all be three distinct nationalistic identities that are embedded through a socializing process within the United States. This articulation of self-identity for various Africana people is not articulated within the framing of Cross’s articulations of nigrescence. Cross’s theory and the U.S. Black Nationalist framing do not readily provide a means for this articulation of identity to be captured within the theory or the implementation of the CRIS. While not providing a substantial platform for this form of nationalism, nigrescence does engage the dualism that is found in a *convergent nationalism*—the dual nationalistic perspectives of hyphenated Americans, emphasizing their nationalistic identity from their respective diasporan identity, linking it with the nationalistic identity that is being “American.” ( De Walt 2009,2011 ). These factors further support the need of an identity model that shifts from usages of race and ethnicity towards that of culture as a way to capture and/or acknowledge the cultural components found within diverse Africana identities.

In recognizing these differences, it also becomes imperative that Universities recognize the evolving demographic of students who stretch traditional conceptions of Blackness or African American identity. As these numbers increase within these contexts, the demand for university services will also increase beyond just superficial activities that are held during particular times of the year. We will need to take a true multicultural approach to the diversity found among various community groups as well those variants found within particular identified groups. This work is daunting but critical to meeting the needs of an ever changing society and set of learners.

## End note

^a^ When referring to a diverse community that is not solely based on cultural identity and race but also speaks to being inclusive of diverse perspectives and ways of knowing.

^b^ While gender and sexual orientation discussions did not permeate through the interactions with the social actors, these aspects of identity are still important and need to be equally considered within university policy.
